# The relationship between HIV infection and depression and their determinants in the MSM population in Eastern China: an analysis based on decision tree modelling and logistic regression

**DOI:** 10.1186/s40359-025-03881-9

**Published:** 2026-01-02

**Authors:** Yiwei Zhou, Zejie Zhang, Wancang Li, Lite Zeng, Chunyan Shan, Tianquan Chen, Zu-Mu Zhou

**Affiliations:** 1 Business School, University of Shanghai for Science and Technology, Shanghai, 200093 China; 2 School of Intelligent Emergency Management, University of Shanghai for Science and Technology, Shanghai, 200093 China; 3 Smart Urban Mobility Institute, University of Shanghai for Science and Technology, Shanghai, 200093 China; 4 Qingtian Center for Disease Control and Prevention, Zhejiang, 323900 China; 5 Department of Health Assessment, Wenzhou Center for Disease Control and Prevention, Wenzhou, 325000 China; 6 Zhejiang Jerinte Health Technology Co.,Ltd, Wenzhou, 325000 China; 7 The Affiliated Kangning Hospital of Wenzhou Medical University Zhejiang Provincial Clinical Research Center for Mental Disorders, 1 Shengjin Road, Huanglong Residential District, Wenzhou, 325007 China

**Keywords:** HIV, Depression, MSM population, Decision tree modelling, Logistic regression

## Abstract

**Objective:**

This study aims to investigate the depression status of the MSM population, explore the depression characteristics associated with different HIV statuses within this group, and determine the key factors influencing depression in this population.

**Study design:**

This was a cross-sectional web-based survey.

**Methods:**

This study was conducted through a web-based comprehensive HIV service platform for the MSM population. The questionnaire included basic socio-demographic information of the respondents, HIV-related information and the Patient Health Questionnaire (PHQ-9). A decision tree model combined with logistic regression was used to assess the factors influencing depression in the MSM population.

**Results:**

Among the 1070 MSM in this study, the depression prevalence was 28.9%, the overall HIV prevalence among MSM was 3.4%, the depression prevalence among HIV-positive MSM was 44.4%, and that among HIV-negative MSM was 26.3%. Decision tree modelling combined with logistic regression analysis showed that marital status, age and employment status were the factors significantly influencing depression in the MSM population.

**Conclusions:**

The prevalence of depression is high in the MSM population. Marital status, age and employment status are influential factors for depression in the MSM population. Decision tree modelling combined with logistic regression could be considered as an assessment tool for the relationship between HIV infection and depression and their correlates in the MSM population.

**Supplementary Information:**

The online version contains supplementary material available at 10.1186/s40359-025-03881-9.

## Introduction

Men who have sex with men (MSM) population are at high risk of HIV infection and sexually transmitted diseases (STDs), and they tend to experience more severe mental health problems than the general population [1–5]. The low social acceptance of MSM, coupled with social discrimination and stigma, as well as the high risk of STDs, are often associated with significant psychological pressure, leading to emotional problems such as anxiety and depression [6, 7]. Chronic depressive symptoms not only cause physical harm, but also increase the incidence of self-harm and suicidal behaviour [7]. The prevalence of depression in MSM is often higher than in the general population [4, 5]. For instance, studies have shown that the prevalence of depression among MSM reaching 54% in Nepal [8, 9], 45.1% in India [10], and is three times higher in in the Netherlands compared to heterosexual men [2]. In the United States, depression rates among MSM have been reported as 33% in Massachusetts [11], 23.1% in Chicago [12] and 22.8% in Seattle [13]. Additionally, MSM have 3–5 times higher rates of depression than the general adult male population. Depression among MSM is a major public health concern, particularly due to its association with risky sexual behaviour and HIV infection [2].

Depression is a major health problem among HIV-positive MSM and is characterized by a persistent low mood or loss of pleasure or interest in activities [14]. Patients with depression experience psychological distress, loss of pleasure, feelings of guilt, disturbances in sleep and eating, and physical fatigue. These symptoms can be long-lasting or recurrent, severely affecting an individual’s studies, work, and daily life, among other things [14]. MSM often face discrimination and stigma from society, family and the workplace. Low family acceptance of their sexual orientation [10], bullying and rejection in social settings, lack of support from friends, family and community in accessing mental health services further increase their risk of depression. This leads to higher rates of depression among this population and potentially serious consequences.

There is a strong association between human immunodeficiency virus (HIV), depression and MSM [14, 15]. Engaging in high-risk sexual behaviors, having multiple sexual partners, inconsistent use of protection, low HIV testing rates, inadequate education and awareness, and the presence of other sexually transmitted infections (STIs) can all contribute to an increased risk of HIV transmission and higher levels of depression among the MSM population [2, 16, 17].

While some research has been conducted on HIV infection in the MSM population and on the psychological status of HIV-positive MSM [2, 18, 19], the psychological status of the MSM population may differ by HIV infection status. There is limited research on depression and its influencing factors among different HIV infection statuses in the MSM population. This study aims to investigate the prevalence of depression in the MSM population, the depression status of MSM by HIV infection status, and the factors influencing depression. The findings will provide valuable insights for the local government and relevant departments to develop appropriate strategies to reduce the prevalence of depression in the MSM population, promote their mental health, and reduce the incidence and spread of HIV/AIDS and sexually transmitted diseases.

## Methods

### Study participants

This study was a cross-sectional survey conducted from March to June 2024 in Eastern China. Given the privacy-sensitive nature of the MSM community, the survey was conducted anonymously via the Internet. The questionnaire was disseminated through an Internet-based comprehensive service platform for HIV prevention and control tailored for the MSM population. To ensure broad coverage and targeted reach, the survey was widely distributed via social platforms such as WeChat groups and QQ groups within the MSM community. Data collection was facilitated through the Golden Data website (www.jinshuju.com). To enhance participation, an incentive policy was implemented to encourage WeChat user to complete the questionnaire. Upon questionnaire completion, each participant received an online gift worth approximately 10 CNY. Each IP address was permitted to submit the questionnaire only once to prevent duplicate entries, and all responses were collected anonymously. Six trained professionals were assigned to oversee data collection and implement quality control measures. Any submitted data that were incomplete or contained logical errors were excluded from the analysis.

### Inclusion and exclusion criteria

The inclusion criteria for this study were as follows: (1) men who have sex with men; (2) age ≥ 18 years; (3) ability to read and complete the questionnaire; (4) proficiency in operating mobile phones; and (5) provision of informed consent and voluntary participation. Exclusion criteria: Participants with incomplete responses or logical errors in their answers were excluded.

### Study instruments

The questionnaire was designed by the researchers themselves and comprised three parts: basic socio-demographic information of the respondents, HIV-related information and the Patient Health Questionnaire (PHQ-9). Basic personal information includes age, occupation, marital status, education, personal income, religious beliefs, employment status, and place of residence. HIV-related information covered HIV test status, syphilis test status, hepatitis C test status, anti-HIV medication status, sexual behavior with men and women, and HIV disclosure status. For more details, see the supplementary file. The PHQ-9 is a widely utilized assessment tool consisting of nine items, each scored on a four-point scale ranging from 0 to 3, with the total score ranging from 0 to 27. Scores of 0–4 were classified as no depressive symptoms, 5–9 as mild, 10–14 as moderate, 15–19 as moderate-severe, and 20–27 as severe. For the purpose of this study, a score of ≥ 10 was used as the cut-off for possible depressive symptoms. The scale has good reliability and validity [20].

### Statistical methods

SPSS 26.0 software was used for statistical analysis. Count data were expressed as the number of cases (percentage), and the chi-squared test was used for intergroup comparisons. Binary logistic regression was used to analyse the factors associated with depression in the MSM population, and variables that were significant in the univariate analyses were included in the logistic regression analyses and decision tree modelling. Statistical inference was performed using the two-tailed test, with *P* < 0.05 as a statistically significant difference.

### Decision tree model

To identify determinants of depression in the MSM population, we applied the Chi-square Automatic Interaction Detector (CHAID) decision tree model, entering depression as the dependent variable and all statistically significant factors from the univariate analysis as the independent variables. CHAID decision tree model was preferred because it efficiently handles categorical variables, exposes interaction effects, and generates an intuitive tree diagram that dovetails with logistic regression, offering a clear, complementary view of how risk factors combine to shape depressive symptoms. The parameters of the CHAID decision tree model were set as follows: the significance level of the split nodes and merged categories of the decision tree was set at 0.05. The growth depth of the decision tree was 3 levels, and the minimum number of cases for the parent and child nodes were 50 and 30, respectively; if the sample size of a node did not meet the minimum requirement, the node became the terminal node and was not further split.

### Ethical review

This study was reviewed and approved by the Ethics Committee of the Affiliated Kangning Hospital of Wenzhou Medical University (Grant No. 2023014). The research was conducted in line with the Declaration of Helsinki and Good Clinical Practice. Before conducting the investigation, each respondent was informed that the information collected during this survey would be kept confidential and would not be disclosed; The data was kept by a special person. Informed consent to participate was obtained from all of the participants in the study. If informed consent was not obtained from the respondents, they could not participate in the survey.

## Results

### Participant characteristics

A total of 1 113 MSM were recruited for this survey, 43 questionnaires with incomplete or logical errors were excluded, 1070 individuals met the inclusion criteria, giving a valid response rate of 96.1%. The age of the respondents ranged from 18 to 65 years, with a mean of (28.5 ± 8.5) years, of which 9.0% were 19 years old or younger (96/1 070); 79.3% were unmarried (848/1 070); 12.8% had an educational level of junior high school or lower (137/1 070); 15.2% had a monthly income of ≤ 1 999 yuan (163/1 070); and 64.6% had no religious beliefs. In terms of employment, 28.7% (307/1 070) were unemployed and 66.3% (709/1 070) lived in urban areas. Regarding sexual behavior in the last six months, 62.1% (664/1 070) had sex with men, and 12.1% (129/1 070) had sex with women. In terms of testing results, 0.4% (4/1 070) tested positive for hepatitis C; 2.4% (26/1070) tested positive for syphilis; and 3.4% (36/1 070) tested positive for HIV. For more details, please refer to Table 1.

**Table 1:** Univariate analysis of factors influencing depression in the MSM population

Variables	Non-depression	Depression	Χ^2^	*P*
Age, years				
18–19	60	36	26.893	˂0.0001
20–29	390	189		
30–39	203	70		
40–49	73	10		
≥ 50	35	4		
Occupation				
Administrative clerks	89	26	4.891	0.299
Agriculture, forestry, fisheries and transport staff	63	19		
Commercial services	109	53		
Professionals	120	50		
Others	380	161		
Marital status				
Unmarried	578	270	17.648	˂0.0001
Married	152	31		
Divorced and widowed	31	8		
Education background				
Junior high school or below	100	37	5.844	0.119
High School	144	45		
College	200	101		
University or above	317	126		
Monthly income, CNY				
≤ 1999	106	57	8.780	0.032
2000–3999	103	55		
4000–5999	252	97		
≥ 6000	300	100		
Religious belief				
Buddhism	187	69	6.369	0.272
Christianity	51	32		
Catholicism	10	5		
Islam	4	2		
Others	11	8		
No Religion	498	193		
Whether employment				
Unemployed	191	116	16.628	˂0.0001
Employed	570	193		
HIV testing				
HIV(--)	640	228	15.682	0.001
HIV (+)	20	16		
Never tested	80	51		
Unwilling to tell test results	21	14		
Whether taking anti-HIV medication				
No	742	289	9.891	0.007
Yes	19	20		
Whether syphilis tested				
(–)	557	207	4.756	0.191
(+)	19	7		
Never tested for syphilis	162	84		
Unwilling to tell test results	23	11		
Whether HCV tested				
(–)	560	198	11.386	0.010
(+)	3	1		
Never tested for HCV	175	92		
Unwilling to tell test results	23	18		
Sexual activity with men in the last 6 months				
No	286	120	0.146	0.702
Yes	475	189		
Sexual activity with woman in the last 6 months				
No	664	277	1.184	0.276
Yes	97	32		
Disclosure of HIV test results				
Self-knowledge only	460	172	10.217	0.037
Family member knows	32	20		
Friends know	189	70		
Others	80	47		
Place of residence				
Rural area	251	110	0.673	0.412
Urban area	510	199		

### Univariate analysis of factors associated with depression in the MSM population

Among the 1070 MSM, 309 (28.9%) exhibited depressive symptoms. Univariate analysis revealed that the prevalence of depressive symptoms in the MSM population was statistically significantly different in relation to age, marital status, income, employment status and HIV testing, use of anti-HIV medication, hepatitis C testing and disclosure of HIV test results (*p* < 0. 05). In contrast, the prevalence of depression did not show statistically significantly difference with respect to occupation, education, religion, syphilis testing, sex with men in the last 6 months, sex with women in the last 6 months and place of residence (*P* > 0.05). See Table 1.

### Logistic regression analysis of factors associated with depression in the MSM population

Eight variables were selected for logistic regression analysis based on their statistical significance in the univariate analysis: age, marital status, income, employment status, HIV test status, use of anti-HIV medication, hepatitis C test status, and disclosure of HIV test results. The results indicated that age and employment status were independent factors influencing depression in the MSM population. Specifically, the odds ratio (OR) for depression in individuals aged 40–49 years compared with ≤ 19 years was 0.347 (95% CI 0.142–0.846), indicating a lower risk of depression in the 40–49 age group. Additionally, the OR for depression in employed individuals compared to unemployed individuals was 0.572 (95% CI 0.394–0.831), suggesting that employment was associated with a lower risk of depression than unemployment. See Table 2.

**Table 2:** Logistic regression analysis of factors influencing depression in the MSM population

Variables	β	S.E	Wald	Significance	Exp(B)	95% CI of EXP(B)	
						Lower	Upper
Age, years							
≤ 19			10.494	0.033			
20–29	0.068	0.250	0.074	0.785	1.071	0.655	1.749
30–39	−0.172	0.289	0.353	0.552	0.842	0.478	1.483
40–49	−1.060	0.455	5.419	0.020	0.347	0.142	0.846
≥ 50	−1.150	0.624	3.391	0.066	0.317	0.093	1.077
Marital status							
Unmarried			1.580	0.454			
Married	−0.319	0.254	1.576	0.209	0.727	0.442	1.196
Divorced and widowed	−0.140	0.449	0.097	0.755	0.869	0.360	2.098
Monthly income, CNY							
≤ 1999			1.807	0.613			
2000–3999	0.296	0.257	1.327	0.249	1.345	0.812	2.226
4000–5999	0.301	0.253	1.415	0.234	1.351	0.823	2.219
≥ 6000	0.211	0.261	0.657	0.418	1.235	0.741	2.060
Employment status: Employed	−0.558	0.190	8.611	0.003	0.572	0.394	0.831
Whether HIV tested							
(–)			1.868	0.600			
(+)	0.228	0.497	0.211	0.646	1.256	0.475	3.325
Never tested	0.130	0.273	0.227	0.634	1.139	0.667	1.947
Unwilling to tell test results	0.518	0.409	1.603	0.205	1.679	0.753	3.744
Whether taking anti-HIV medication							
No			4.413	0.110			
Yes	0.810	0.465	3.027	0.082	2.247	0.903	5.596
Whether HCV tested							
(–)			1.810	0.613			
(+)	0.050	1.204	0.002	0.967	1.051	0.099	11.132
Never tested	0.138	0.194	0.508	0.476	1.148	0.785	1.679
Unwilling to tell test results	0.469	0.372	1.588	0.208	1.598	0.771	3.312
Disclosure of HIV test results							
Self-knowledge only			3.491	0.479			
Family member knows	0.214	0.332	0.415	0.520	1.238	0.646	2.371
Friends know	−0.046	0.173	0.071	0.790	0.955	0.680	1.341
Others	−0.264	0.461	0.327	0.567	0.768	0.311	1.896

### Decision tree analysis of factors influencing depression in the MSM population

**Fig. 1:**
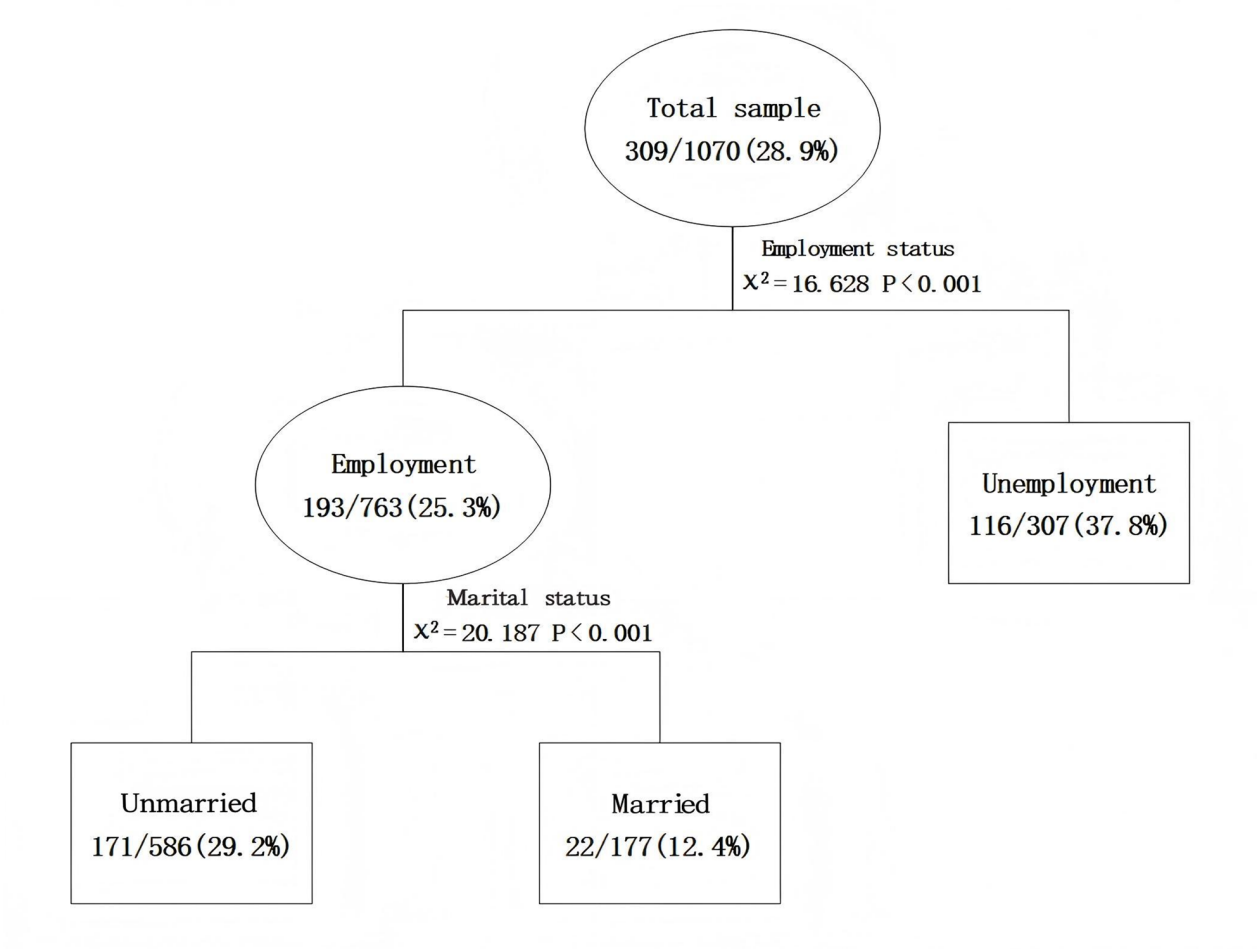
Decision tree of factors influencing depression in the MSM population

The CHAID decision tree model revealed that employment status and marital status significantly influenced depression occurrence in MSM. The first decision tree layer showed that unemployment was associated with a higher depression rate (37.8%) than employment (25.3%) (χ² = 16.628, *P* < 0.001). The second layer highlighted marital status as another key factor. Among employed MSM, those who were unmarried exhibited a higher prevalence of depression at 29.2% compared to their married counterparts at 12.4% (χ² = 20.187, *P* < 0.001). Furthermore, the model indicated an interaction effect between employment status and marital status, as depicted in Fig. 1.

### Prevalence of depressive symptoms in the MSM population by HIV status

Among the 1070 MSM, there were 36 HIV-positive cases, yielding an HIV-positive rate of 3.4%. Of these 36 HIV-positive individuals, 16 (44.4%) exhibited depressive symptoms. In contrast, among the 868 HIV-negative participant, 228 (26.3%) had depressive symptoms. Additionally, of the 35 individuals unwilling to disclose their HIV test results, 14 (40.0%) showed depressive symptoms, and of the 131 individuals who had never been tested for HIV, 51 (38.9%) had depressive symptoms. See Table 3 for details.

**Table 3:** Prevalence of depressive symptoms in the MSM population by HIV status

variables	Number of case	Non-depression	depression	prevalence (%)
HIV (+)	36	20	16	44.4
HIV (-)	868	640	228	26.3
Never HIV tested	131	80	51	38.9
Unwilling to disclose their HIV test results	35	21	14	40.0
Total	1070	761	309	28.9

## Discussions

Depression is more prevalent among MSM than in the general population in both developed and developing countries [1], yet its prevalence varies across regions [2, 11, 21]. In the U.S. and Australia, depression rates among MSM from 22.8% to 33% [2, 11], while in France, depression prevalence was 28.1% among MSM [22]. Even within the U.S., rates also vary by state, ranging from 22.8% in Seattle and 23.1% in Chicago to 33% in Massachusetts [11–13]. The Tanzanian study found a prevalence of depression among MSM of 46.3% [2] and two studies in South Africa reported prevalence rates of 44% and 56% [2, 21, 23]. Our survey found a 28.9% depression rate among MSM, lower than in Tanzania, South Africa, and Australia [2, 11] but higher than in Chicago and Seattle [2, 11], and similar to the prevalence observed in France [22]. This heterogeneity signals that locally salient stressors—rather than a universal minority stress—drive poor mental health. Tailoring interventions therefore requires unpacking context-specific risk profiles instead of importing “one-size-fits-all” programmes.

Our decision-tree analysis identified two modifiable nodes that increased depression risk locally: unemployment and being unmarried. Embedding these findings into an operational definition of ‘effectiveness’ strives to meet international standards while considering local realities. Consistent with criteria used in nutrition-behaviour trials and physical-activity interventions for socially isolated older adults [24, 25], we define an effective anti-depression intervention for MSM as one that, relative to control or baseline: (1) achieves a statistically significant and clinically meaningful improvement (≥ 6-point PHQ-9 reduction [26], 20% reduction in PHQ−9 scores [27], or Cohen’s d ≥ 0.3) [28]; and (2) is acceptable, scalable and sustainable in the setting where it will run. Translating this definition into practice, eastern-China health authorities could integrate three low-cost, high-feasibility components into the existing digital HIV-prevention ecosystem: (1) Embed a two-minute PHQ-9 screen into the HIV-testing apps already ubiquitous among local MSM, ensuring seamless uptake without additional download burden. (2) Upon simultaneous completion of HIV testing and the depression scale, instantly issue an appropriate number of small-denomination cash top-up vouchers (e.g., ¥20) or e-coupons for vocational-training courses, directly offsetting the unemployment pathway while incentivising completion. (3) Push time-limited WeChat-mini-program coupons for free or subsidised counselling that is explicitly branded “partner-friendly”, reducing the help-seeking barrier faced by unmarried men who lack spousal encouragement. All elements leverage the region’s near-universal smartphone ownership and the same platform that hosted our survey, guaranteeing rapid uptake, cultural congruence and immediate action on the risk factors our model uncovered. By anchoring effectiveness benchmarks to internationally recognised thresholds while delivering the intervention through locally embedded, app-based touch-points, we reconcile global rigour with contextual relevance—an alignment that, according to our data, is indispensable if the mental-health gap among Chinese MSM is to close.

Employment status, marital status and age have a significant impact on depression in the MSM population in our study. Several studies indicate unemployment can increases stress, anxiety and even depression [29, 30]. The present study show that the prevalence of depression was significantly higher among the unemployed than among the employed, which is consistent with the above findings [29, 31, 32].

The effect of marital status on depression is variable [33, 34]. Some studies suggest that married individuals may be more prone to depression than unmarried one [2]. For instance, Yang et al. [33] reported that currently married MSM had the highest depression symptoms compared to single and divorced individuals. However, other studies indicated that married MSM has not a higher prevalence of depression than, single or divorced people [34]. In our study, being married was a significant determinant associated with higher depression in the MSM population. These discrepancies may stem from differences in study periods, sites, population, subgroup compositions, and measurement tools [35].

The present study shows that the risk of depression was lower in those aged 40–49 years than in those ≤ 19 years, which is consistent with the report by Thirunavukkarasu et al. [10, 33] but is not consistent with other studies [34, 36]. The reasons for the different results need to be further investigated in the future.

While numerous studies have reported the prevalence of depression in HIV-positive MSM populations [14, 37–39], comparatively fewer have focused on the prevalence of depression in HIV-negative MSM populations. Nouri et al. [35] reported a 47% prevalence of depression among 3 753 HIV-positive MSM in 11 studies, compared with a 33% prevalence among 47 788 HIV-negative MSM in 60 studies. The prevalence of depression was significantly higher among HIV-infected MSM than among HIV-uninfected MSM. Among the 1 070 MSM in our study, the overall HIV prevalence was 3.4%. Among HIV-positive individuals, 44.4% were depressed, compared with 26.3% of HIV-negative individuals. Although HIV-positive MSM had a significantly higher rate of depression than HIV-negative individuals in unadjusted analysis, this association was not significant after adjusting for covariates. This finding does not aligns studies indicating that HIV-positive MSM in sub-Saharan Africa have higher depression rates [14, 40, 41]. This discrepancy may be due to regional differences in social determinants of health, access to healthcare, or study methodologies. Thus, enhanced social support and psychological counseling for MSM, regardless of HIV status, are crucial to reducing depression. Implementing targeted interventions that address the specific needs of MSM and promoting mental health literacy can contribute to improved mental health outcomes in this population.

Decision tree algorithms and logistic regression are widely used clinically for the prediction and analysis of various disease risk factors [42–47]. Decision tree, as a machine learning method, is intuitive use of probabilistic analysis of a graphical method. It is easy to implement, sensitive to the data analysis, interpretable, and can be visualised and analyzed, fully aligning with human intuitive thinking, and thus having a wide range of applications. However, the decision tree model is rarely used for analyzing depression-related factors. Furthermore, after searching several databases such as PubMed, no studies were found that used the decision tree algorithm for predicting or analyzing depression risk factors in the MSM population. In this study, the Chi-Square Automatic Interaction Detector (CHAID) decision tree algorithm combined with a logistic regression model was used to analyse the factors associated with depression in the MSM population.

Our finding highlights the complementary strengths of using both logistic regression and decision tree models in analyzing depression among MSM. The logistic regression identifies age and employment status as key independent factors influencing depression, underscoring the importance of these variables in traditional statistical analysis. Meanwhile, the CHAID decision tree model not only corroborates the significance of employment status but also reveals marital status as an additional influential factor. This suggests that the decision tree model can capture nuances in the data that logistic regression might miss, possibly due to its ability to handle non-linear relationships and interactions between variables more flexibly. The fact that marital status emerged as a factor in the decision tree but not in the logistic regression could indicate that its effect is more complex or context-dependent, and might be better uncovered through the decision tree’s partitioning approach. Overall, this demonstrates how combining these analytical methods can provide a more comprehensive understanding of the factors associated with depression in the MSM population, potentially leading to more effective and targeted interventions.

### Limitations

This study has the following limitations: First, this is a cross-sectional survey. As such, the data were collected at a single point in time, which means the results can only show associations and cannot establish causality. Second, depressive symptoms and HIV status were self-reported by the study participants. Self-reported data can be subject to biases. Participants might not remember accurately or might be hesitant to report certain information due to social stigma or other concerns. This could lead to information bias, where the data might not fully reflect the true situation. Third, due to the sensitive and privacy nature of the MSM population, some potential influencing factors were not included in this study. For instance, detailed information on specific behaviors or social contexts that could influence the outcomes was not collected. This limitation means that our findings might not capture all the important factors at play. Overall, while our study provides valuable insights, these limitations should be considered when interpreting the results. Future research could benefit from longitudinal designs, more comprehensive data collection methods, and additional validation of self-reported information to address these issues.

## Conclusion

This study examined the depression status of the MSM population, explored the depression characteristics associated with different HIV statuses within this group, and applied decision tree modelling and logistic regression to assess the factors influencing depression. Among the 1070 MSM studied, the depression prevalence was 28.9%, with an overall HIV prevalence was 3.4%. The decision tree algorithms combined with logistic regression modelling showed that marital status, age and employment status were the factors influencing depression in this population. The combined use of the two methods can complement each other and improve the analytical effect. These findings provide a scientific basis for the government and relevant departments to develop targeted measure. These findings provide a scientific basis for governments and relevant departments to develop targeted measures. Such measures can help reduce depression rates in the MSM population, promote their physical and mental well - being, and lower the incidence of STDs and AIDS.

## Supplementary Information

Supplementary Material 1.

## Data Availability

The datasets used and analyzed in this study are available from the corresponding author on reasonable request. Because of the sensitive nature of the data collected on the mental health of MSM amongst which individuals are potentially identifiable, we cannot provide open access to our data.

## Abbreviations

STD: Sexually Transmitted Disease
STI: Sexually Transmitted Infection
AIDS: Acquired Immunodeficiency Syndrome
HIV: Human Immunodeficiency Virus
MSM: Men Who Have Sex With Men
OR: Odds Ratio
CI: Confidence Interval
SPSS: Statistical Package for the Social Sciences
CHAID: Chi-Square Automatic Interaction Detector
PHQ-9: Patient Health Questionnaire-9
