# Supplementary material for: The relationship between HIV infection and depression and their determinants in the MSM population in Eastern China: an analysis based on decision tree modelling and logistic regression
Source: BMC Psychol. 2026 Jan 2;14:146. doi: 10.1186/s40359-025-03881-9 (PMC12866518; doi:10.1186/s40359-025-03881-9)
Supplement: Supplementary file 1 — Supplementary Material 1. [file 40359_2025_3881_MOESM1_ESM.pdf]

Depression Screening and Background Information Questionnaire

1. Age:
2. Occupation:
   - Administrative staff
   - Professional/technical personnel
   - Clerical and related workers
   - Business and service workers
   - Agricultural, forestry, animal-husbandry, fishery or water-industry workers
   - Production/transport-equipment operators
   - Other

3. Marital status:
   - Married - Never married - Divorced - Widowed - Cohabiting

4. Education:
   - Below junior high - Senior high - College / technical school - University and above

5. Personal monthly income (CNY):
   - < 2 000 - 2 000 – 3 999 - 4 000 – 5 999 - ⩾ 6 000

6. Religion:
   - Buddhism - Protestant Christianity - Catholicism - Islam - Other religion
   - None

7. Employment status:
   - Stable job - No stable job

8. HIV test result:
   - Negative - Positive

   If positive, date of diagnosis: month___ day ___ year___ (____ months ago)

9. Currently taking anti-HIV medication: Yes / No

10. Syphilis test result: Negative - Positive - Don't know - Not tested

11. Hepatitis B test result: Negative - Positive - Don't know - Not tested

12. Hepatitis C test result: Negative - Positive - Don't know - Not tested

13. In the past 6 months:

- Sex with men: Yes / No
- Sex with women: Yes / No

14. HIV disclosure:
   - Family aware - Friends aware - Other (specify) ___________

15. Residence: Rural - Urban

16. Patient Health Questionnaire-9 (PHQ-9)

Over the last 2 weeks, how often have you been bothered by any of the following problems? Please choose the answer that best fits your actual situation.

Item	Not at all	Several days	More than half the days	Nearly every day
1.Little interest or pleasure in doing things	0	1	2	3
2.Feeling down, depressed, or hopeless				
3.Trouble falling or staying asleep, or sleeping too much				
4.Feeling tired or having little energy				
5.Poor appetite or overeating				
6.Feeling bad about yourself - or that you are a failure or have let yourself or your family down				
7.Trouble concentrating on things, such as reading the newspaper or watching television				
8.Moving or speaking so slowly that other people could have noticed. Or the opposite - being so fidgety or restless that you have been moving around a lot more than usual				
9.Thoughts that you would be better off dead, or of hurting yourself in some way				
